# Imaging of Neurovascular Activity during Motor Responses Evoked by Displacement-guided Focused Ultrasound (DgFUS) Neuromodulation in Mice

**DOI:** 10.21203/rs.3.rs-9173358/v1

**Published:** 2026-04-23

**Authors:** Seongyeon Kim, Daniella A. Jimenez, Jonas Bendig, Moshe J. Wilner, Gillian L. Ciaccio, Sergio Jiménez-Gambín, Nancy Kwon, Saachi Munot, Erica P. McCune, Samuel G. Blackman, Elisa E. Konofagou

**Affiliations:** 1 Department of Biomedical Engineering, Columbia University, New York, NY, USA; 2 Department of Neurology, Columbia University, New York, NY, USA; 3 Department of Radiology, Columbia University, New York, NY, USA

**Keywords:** Displacement-guided focused ultrasound (DgFUS), Functional ultrasound (fUS) imaging, Neurovascular response, Cerebral blood volume (CBV), Electromyography (EMG)

## Abstract

Focused ultrasound (FUS) shows great promise for precise and non-invasive neuromodulation, with applications from basic neuroscience investigation to the treatment of neurological disorders. Yet, mechanistic monitoring of FUS is challenging and a causal link between FUS-induced displacement and both neurovascular and behavioral responses evoked by FUS in mice remains poorly understood. Here we harness displacement-guided focused ultrasound (DgFUS) to induce target-specific and non-auditory motor responses in anesthetized mice, and functional ultrasound (fUS) to image neurovascular activity during the evoked responses. We show that DgFUS elicits ipsilateral neurovascular responses, in which the primary (i.e. focal-onset) activation localizes with in situ brain displacement, followed by mild contralateral responses. We demonstrate that switching the target hemisphere of DgFUS within the thalamus results in lateralized hindlimb responses concurrent with lateralized neurovascular activation. We show strong correlation between displacement and both cerebral blood volume (CBV) and electromyography (EMG) measurement (Disp. vs. CBV: Spearman r = 0.9091, p < 0.0001, Disp. vs. EMG: r = 0.7332, p = 0.0091, CBV vs. EMG: r = 0.9041, p = 0.0002), indicating displacement-driven mechanism by which DgFUS evokes neurovascular and motor responses. Lastly, we demonstrate anxiety-related behavioral improvement with transcranial fUS-guided FUS delivered to the bilateral hippocampus, consistent with increases in resting-state hippocampal connectivity assessed by transcranial fUS imaging. The findings presented herein will help refine our understanding of neurovascular responses to FUS and the consequential motor and cognitive outcomes.

## 1 Introduction

Focused ultrasound (FUS) is a promising technique toward modulation of excitatory and inhibitory neurons, showing immense potential for studying brain function and tackling neurological diseases [1, 2, 3]. Advances in concurrent neuroimaging techniques and image guidance of ultrasonic dose have significantly augmented the capability of FUS and facilitated precise neuromodulation with high spatial resolution and deep penetration depth. Displacement-guided focused ultrasound (DgFUS) enables targeted neuromodulation in the central (CNS) [4] and peripheral nervous system (PNS) [5, 6, 7, 8] by tracking *in situ* micron-level displacement induced by FUS at a sub-millisecond scale with a confocal ultrasound imaging array.

To fully harness FUS neuromodulation, it is important to understand hemodynamic responses that reflect neural activity evoked by FUS (i.e. neurovascular responses) and to correlate the evoked responses with motor and cognitive outcomes. Several studies have demonstrated FUS-evoked motor responses in mice [9, 10, 11], but lacked evidence of target-specific and non-auditory responses to FUS. The demonstration was often limited to tail responses without precise control of other body parts such as paws and limbs, sometimes accompanied by whole body movement and no substantial latency that may imply startle reflex associated with auditory pathway [12, 13] and/or arousal/pain-related responses. Notably, [14, 15] demonstrated precise motor control that included lateralized hindlimb movement by switching sonicated hemispheres, indicating highly target-specific and non-auditory motor responses to FUS. However, all of the aforementioned studies lack simultaneous neuroimaging with FUS. The FUS-evoked responses are highly dependent on brain targets, FUS parameters, and types of anesthetics [1]. Thus, concurrent mechanistic monitoring is a key to resolve discrepancies across different trials and to inform neuromodulation outcome.

Ultrafast Doppler and super-resolution imaging techniques have emerged over the past decade and showed great promise to explicate brain function with a superior spatiotemporal resolution [16, 17]. We previously harnessed functional ultrasound (fUS) imaging simultaneously with FUS and demonstrated localized FUS-evoked hemodynamic responses in mice under isoflurane [4]. Beyond this report, several studies have demonstrated ongoing efforts towards identifying localized brain response to FUS across species, ranging from mice [18, 19, 20] to a human [21]. However, most preclin- ical studies lack a behavioral readout and a correlation with brain responses evoked by FUS. Thereby, mechanistic monitoring of FUS and linking neurovascular activity and conseqential motor responses evoked by FUS remain open challenges.

To address the existing challenges in previously reported studies, we hereby imaged neurovascular activity with fUS imaging while delivering DgFUS to elicit targetspecific and non-auditory motor responses. We elicit localized neurovascular and target-specific motor responses using a single 80-ms continuous FUS pulse with a significantly less ultrasonic dose compared to our previous studies [4, 14], which is aligned with more safe, translatable approach and conducive to the mechanical effect of FUS. By switching sonicated hemispheres with DgFUS, we demonstrate DgFUS effectively lateralizes hindlimb responses in mice confirmed by the simultaneously imaged lateralized neurovascular activation. Lastly, we demonstrate anxiety-related behavioral changes in the hippocampus with transcranial fUS-guided FUS delivered to the bilateral hippocampus. We anticipate that our findings may yield image correlates of behavioral outcomes, which can consolidate understanding of neurovascular responses evoked by FUS and its relationship with motor and cognitive outputs.

## 2 Results

### 2.1 DgFUS evokes localized and ipsilateral neurovascular response followed by contralateral neurovascular response at cortical and subcortical depth

First, we sought to characterize DgFUS-evoked neurovascular responses that are associated with motor responses. We performed fUS imaging while delivering DgFUS to evoke hindlimb, hindpaw, toes, and/or tail-related motor activation. Displacement imaging was performed to confirm target validation and to image *in situ* brain displacement induced by FUS. Fig. 3 (A) demonstrates five snapshots of CBV changes at 0, 3, 4, 7, and 23 s after the onset of FUS targeting the right hemisphere at 2.54 MPa that evokes bilateral toe twitches and tail movement (CBV Movie: Supplementary Movie 3A). Corresponding *in situ* brain displacement map was depicted in Fig. 3B. Fig. 3C illustrates a FUS-evoked toe twitch (Supplementary Movie 3B). We observed ipsilateral CBV increases in the cortex and thalamus at 3-4 s post FUS during 10 s stimulation period, which is followed by a contralateral response at 7 s post FUS. To temporally resolve activation map and identify CBV propagation, we applied variable lags to the binary stimuli vectors to compute Pearson’s correlation for the activation map. Fig. 3E depicts the activation map in case of −1, 3, and 5 s lag, and the corresponding binary stimuli vectors were plotted in Fig. 3G. The lag of −1 s accentuates focal-onset CBV engagement and the activation map successfully captured primary CBV changes which is highly ipsilateral, particularly with mild activation at the somatosensory cortex and robust activation at the thalamus. Importantly, the primary site of subcortical activation was localized with −3dB *in situ* brain displacement map. With positive and larger lags, the activation pattern changed, and cortical activation became much pronounced, which is followed by the contralateral activation. These observations may indicate that FUS initiated neurovascular activation at the thalamus directly, which in turn propagated to the cortical area via an upstream (or bottom-up) thalamocortical pathway [22, 23], leading to hindpaw and tail responses. To trace and map neurovascular activity that is causally linked to toe twitch, we collected CBV signals in response to electrical stimulation (1.5 mA, 7 ms, 4 Hz PRF, 20 s) that evoked toe twitches in the left hindpaw in mice. Fig. 3D depicts the activation area elicited by electrical stimulation (CBV trace shown in Supplementary Fig. S3). Neurovascular responses to electrical stimulation were observed in the somatosensory cortex, along with consistent FUS-evoked neurovascular activation in the somatosensory cortex (Fig. 3E, center). These results strongly support the causal link between FUS-evoked neurovascular activation in the cortical areas and motor responses, particularly those related to toes and hindpaw.

Fig. 3F depicts CBV responses at the thalamus (left) and somatosensory (right) and corresponding ROIs are illustrated in Fig. 3E. We found that FUS induced CBV increases with a peak CBV amplitude of 15.45% ± 6.1% and 13.86% ± 6.9% at the ipsilateral cortex and thalamus at 2.54 MPa, respectively, which is followed by the contralateral CBV changes with a lower peak CBV. To compare the ipsilateral and contralateral responses, two-tailed paired t-test was performed on CBV responses at 3.39 MPa (n = 6 animals, Fig. 3I). The quantification was performed based on CBV at the thalamus that is the primary site of CBV activation observed. Our results indicate stronger and faster CBV activation in the ipsilateral hemisphere with significantly lower peak latency (p = 0.0422), higher peak CBV changes (p = 0.0237), and larger activation area (p = 0.0022), compared to the contralateral hemisphere. Maximum correlation coefficient showed good agreement with the stronger ipsilateral activation but did not reach statistical significance (p = 0.0509). In addition, we evaluated the size of the activation area as a function of lag and compared the ipsilateral and contralateral activations. We found that the size of the ipsilateral activation area increases faster than the size of the contralateral activation area (Fig. 3H), which is consistent with the aforementioned findings on the latency. In addition, we found that group-averaged CBV responses were dose-dependent. (Supplementary Fig. S2).

### 2.2 DgFUS evokes target-specific and dose-dependent motor responses that correlate with neurovascular response

A single continuous 80 ms FUS pulse successfully elicited motor activation in ketamine/xylazine (K/X) anesthetized mice. Fig. 4A depicts compound muscle action potential (CMAP) evoked by FUS at 3.39 MPa (left; collected from tail muscle) and root mean square (RMS) envelope of rectified signals (right) after processing (see Methods). Specifically, we observed lateralized hindlimb/paw movement (Supplementary Movie 4A and 4B) and/or toe twitches with tail movement vigorously at 2.54 and 3.39 MPa (Fig. 4C). Fig. 4B demonstrated normalized RMS signals with increasing pressure. We observed dose-dependent motor responses with a peak EMG amplitude increasing with pressures (Fig. 4D). No significant motor responses were observed in sham (0 MPa) or off-target cases (data provided in Fig. 5E). Peak latencies were found to be 72.42 ± 3.74, 67.24 ± 6.97 and 65.38 ± 8.52 ms (mean ± std, n = 69 stimulations) at 1.69, 2.54, and 3.39 MPa, respectively (Fig. 4E), demonstrating a decreasing trend with increasing pressure which is in line with earlier in vivo study with high frequency FUS (*f_c_* = 5 MHz) stimulation [24]. The latencies with pressures greater than 2.54 MPa were not statistically different (p = 0.238, One-way ANOVA with Tukey correction), both of which were significantly shorter than 1.69 MPa (p < 0.0001). Note that, we did not observe cumulative effects [14] on motor responses during the experiment throughout this study. Additionally, we performed a two-tailed nonparametric Spearman’s correlation and a linear regression between peak EMG amplitude and peak CBV changes (Fig. 4F, 12 CBV/EMG responses from n = 4 animals). The significant correlation (r = 0.9041, p = 0.0002, *R^2^* = 0.6876) indicates that the observed motor responses were mediated by the FUS-evoked neurovascular activation.

### 2.3 Spatially translated 4 MHz DgFUS drives co-localized thalamic activation and directional hindlimb motor activation

Next, we leveraged high spatial specificity of neuromodulation with 4 MHz DgFUS and questioned whether spatially translated DgFUS lateralizes neurovascular activation and causal motor responses. To address this question, we physically translated the confocal configuration of the imaging and FUS transducers by 2-3 mm to alternate between the left and right thalamus and performed fUS imaging simultaneously with FUS neuromodulation (Fig. 5A left). The bottom left panel in Fig. 5A depicts focal displacement induced by left thalamus FUS. Both hindlimbs were tracked using DeepLabCut^™^ [25] (Fig. 5A right) and the left and right hindpaw velocities were computed based on 2D Euclidean distance. Fig. 2B illustrates time traces of the left and right hindpaw velocities induced by the left and right thalamus FUS. We demonstrated lateralized hindpaw movement depending on the targeted hemisphere (Fig. 5C and 5D). Two-tailed unpaired t-test indicates left thalamus FUS selectively induced left hindpaw movement (LH: −81.77 ± 31.14 vs. RH: 19.01 ± 19.9 pixels/ms; p < 0.0001, Supplementary Movie 5A). Conversely, targeting the right thalamus reversed the effect, selectively inducing right hindpaw movement (LH: −0.45 ± 7.69 vs. RH: 20.2 ± 18.46 pixels/ms; p = 0.0069, Supplementary Movie 5B). We demonstrated that sub-millimeter off-target (i.e., FUS positioned forward by 0.5 mm with respect to on-target that evokes bilateral toe twitch) led to no significant motor responses comparable to sham (p = 0.74, One-way ANOVA with Welch correction), indicating that the FUS-evoked motor responses we observed were highly target-specific (Fig. 5E, Supplementary Movie 5C). In addition, physically translated 4 MHz FUS successfully shifted CBV activation area, eliciting highly localized and lateralized neurovascular activation in the thalamus (LTh FUS: Fig. 5F and Supplementary Movie 5D, RTh FUS: Fig. 5G and Supplementary Movie 5E). We observed the ipsilateral hemisphere exhibited significantly stronger CBV changes compared to the contralateral hemisphere (LTh FUS: p = 0.0166, RTh FUS: p = 0.0083), accompanying with larger activation area and higher correlation coefficients. These findings highlight the directional dependence of hindlimb movement on the thalamic activity, which may establish a topographical and causal link between FUS-evoked neurovascular and motor responses.

### 2.4 Displacement spatially and quantitatively correlates with neurovascular and motor responses evoked by DgFUS

We further investigated how *in situ* brain displacement evoked by DgFUS spatially and quantitatively links with neurovascular and motor responses. Fig. 6A depicts an example displacement-activation map with left thalamus FUS. Two-tailed nonparametric Spearman correlation with all pixels within the brain indicates a positive monotonic relationship between displacement and CBV activation (Spearman’s r = 0.4166 and p < 0.0001). The activated pixels (Pearson’s r > 0.2) exhibited significantly greater correlation coefficient compared to non-activated pixels (p < 0.0001, two-tailed unpaired t-test with Welch correction). Our findings demonstrate spatial correlation (i.e. colocalization) between FUS-evoked brain displacement and neurovascular activation. Fig. 6D and 6E depict scatter plots of peak displacement vs. peak CBV changes and peak EMG amplitude, respectively (12 CBV/EMG responses from n = 4 animals). We performed two-tailed nonparametric Spearman correlation and a linear regression. Peak displacement was found to be significantly correlated with both peak CBV changes (r = 0.9091, p < 0.0001, *R*^2^ = 0.869) and peak EMG amplitude (r = 0.7332, p = 0.0069, *R*^2^ = 0.535), indicating that FUS-evoked neurovascular and motor responses we observed in this study were mediated by displacement-driven (i.e. acoustic radiation force-based) mechanism.

### 2.5 Transcranial fUS-guided FUS delivered to the bilateral hippocampus induces anxiety-related behavioral changes

Finally, we sought to explore translational and therapeutic potential of fUS-guided FUS approach. We performed transcranial fUS imaging simultaneously with FUS on mice with intact skull to guide and monitor hippocampal FUS sonication. The left panel in Fig. 7A illustrates an example of a transcranial power Doppler image. We sonicated bilateral hippocampus (ML: ± 2.5, AP: - 2.5, DV: 2 mm) and FUS was applied for 2 minutes (1 Hz PRF, 1.9 MPa pressure, and 150 ms pulse duration) with a total of 120 FUS pulses delivered per each bilateral target. This FUS neuromodulation (FUS-N) regime was established to maximize ultrasonic dose and to facilitate long- lasting effect of ultrasound [26]. For comparison, we have included a mouse group that underwent FUS-mediated blood-brain barrier opening (FUS-BBBO, see Methods). To examine the effects of FUS-N and FUS-BBBO on anxiety and potential neurogenesis effects, we conducted open field test and immunofluorescence analyses three weeks after the sonication (Fig. 7A).

Fig. 7B depicts activation map with FUS-N on the right hippocampus (Tran- scranial simulation: Supplementary Fig. S6). Transcranial fUS imaging confirmed successful neurovascular engagement at the targeted hippocampus that exhibited peak CBV changes of ~ 8.5 % (Fig. 7C). Fig. 7D demonstrates the time in the center zone across the FUS-BBBO, FUS-N (n = 5 animals), and sham mice (n = 4 animals). Fig. 7E demonstrates representative trajectory images from the open field test. The mice in the FUS-N group spent 72.02 ± 23.59 seconds in the center zone. The FUS-BBBO spent 65.97 ± 47.75 seconds while the sham spent 15.00 ± 9.191 seconds in the center zone of the area. One-way ANOVA revealed a significant effect of treatment (p < 0.05). Post-hoc Dunnett’s test indicated that the effect of FUS-N was significant (p = 0.0423), while FUS-BBBO vs. sham did not reach significance (p = 0.0683). Fig. 7F depicts representative images of DAPI and doublecortin positive (DCX+) cells. We found that increasing time in the center zone correlated with increasing number of DCX+ cells for both FUS paradigms. For the FUS-BBBO group, the relationship between DCX count and center zone time was significant (y = 0.3356x + 0.268, *R*^2^ = 0.6878, p = 0.0057). For the FUS-N group, the relationship approached significance (y = 0.2554x + 8.305, *R*^2^ = 0.3923, p = 0.0527). The correlation indicates that neurogenesis may partially contribute to the anxiety-related behavioral changes we observed in the study.

## 3 Discussion

Mechanistic monitoring of FUS neuromodulation is important to elucidate mechanical and biophysical mechanisms by which FUS modulates neural activity and induces consequent motor and cognitive responses and to ensure successful and safe neuromodulation. In this study, we harnessed displacement and fUS imaging to mechanistically monitor the brain while delivering FUS to elicit target-specific motor responses in mice. In addition, we demonstrated anxiety-related behavioral improvement following FUS-evoked neurovascular modulation in the hippocampus guided by transcranial fUS imaging.

We previously introduced displacement and/or fUS-guided FUS system [4, 27] and found that FUS-evoked hemodynamic responses correlate with brain displacement. However, the study lacks target specific FUS-evoked motor responses that our previous studies demonstrated [14, 27], which led us to question whether fUS imaging can detect FUS-evoked CBV responses that truly reflect neuronal activity causally evoking motor activation. Furthermore, temperature rise up to 5.24 °C caused by 300 ms pulse duration could partially contribute to hemodynamic responses we reported in the study [4]. The aforementioned limitations motivated us to pursue mechanistic monitoring of FUS neuromodulation that accompanies with target-specific (i.e. non- auditory) motor response evoked by FUS with a lower ultrasonic dose. Therefore, here in this study, we sought to establish the FUS parameter that induces robust motor activation in a mechanical manner without significant heating, and to perform displacement and fUS imaging of mouse brain through a cranial window during motor responses evoked by FUS neuromodulation.

We found that a single continuous 4 MHz FUS pulse with a pulse duration of 80 ms and a pressure greater than 1.54 MPa is capable to elicit motor activation of hindlimb, hindpaw (including toes or digits), and tail in the K/X anesthetized mice (Fig. 4C). We observed a peak EMG latencies of 65.38 ± 8.52 ms at 3.39 MPa, which shows good agreement with the previous studies reporting FUS-evoked EMG responses [15, 28] and greatly exceeds an expected latency of startle-like reflex evoked by the auditory and somatosensory confounds (~10 msec) [28]. Notably, we discovered toe twitch responses which have not been previously reported with central FUS neuromodulation to our knowledge (Supplementary Movie 3A). The individual toe movement appears very comparable to toe twitches in response to sciatic nerve stimulation with FUS [6, 7]. We believe that evoking thumb twitch with central FUS may be possible due to a high spatial specificity of 4 MHz FUS. We hypothesized that the high spatial resolution of 4 MHz FUS enables to selectively activate sensorimotor pathway signaling through a sciatic nerve to the distal branches that innervate hindpaw and digits, which in turn causally evokes individual toe movement, whereas peripheral FUS activates the sciatic nerve directly and the signal passes down to the distal branches. We believe this interplay or reciprocity between CNS and PNS neuromodulation has yet to be fully investigated and would be of great interest to explore moving forward.

In this study, we administered ketamine and xylazine to sedate mice and evoke locomotor activity during fUS imaging simultaneously with FUS neuromodulation. While isoflurane is predominantly used in preclinical studies on FUS neuromodulation due to its ease of administration, very low-levels of isoflurane (< 0.1%) is conducive to the observed responses [9, 10] that may be indeed startle-like reflex evoked by the auditory and somatosensory confounds [28], rather than non-auditory motor responses that arise from direct activation of motor neural circuits. Ketamine is characterized as a noncompetitive N-methyl D-aspartate receptor (NMDAR) antagonist that interacts with voltage-gated ion channels and synaptic transmission [29]. Several pieces of in vitro and in vivo evidence indicate that ketamine blocks cortical neuron activity and inhibits FUS-mediated NMDAR activation that can contribute to the initiation of Ca2+ reflux [30, 31]. Due to the suppressive effect of ketamine, a modest depth of K/X anesthesia is known to be conducive and allowed monitoring window for FUS-evoked locomotor activities in mice and rabbit [30, 32]. In addition, the mixture of ketamine and xylazine has been widely used and validated as an anesthetic in mouse fMRI studies, demonstrating robust and reliable BOLD signals in response to somatosensory [33, 34, 35, 36] and optogenetic stimulation [35], as well as FUS stimulation [19]. Therefore, in this study, we chose to use K/X anesthesia for mechanistic monitoring of FUS-evoked motor responses under an adequate anesthesia depth that coincides with a mild response to toe pinch and without any significant spontaneous locomotion.

We observed localized, ipsilateral, and dose-dependent CBV increases in response to FUS, which correlates with dose-dependent motor responses quantified by EMG measurement. Our fUS imaging reveals significantly stronger CBV responses in the ipsilateral hemisphere compared to the contralateral hemisphere under K/X anesthesia (mean ± std; ipsi: 30.15 ± 15.47 % vs. contra: 16.70 ± 8.54 %; Fig. 3I), which is consistent with our previous findings on FUS-evoked CBV responses in mice under isoflurane [4] and a recent study with functional optoacoustic tomography demonstrating milder FUS-evoked hemodynamic changes in the contralateral hemisphere [20]. In addition, we found significantly faster CBV engagement in the ipsilateral hemisphere compared to the contralateral CBV response (at 4.33 ± 0.51 s vs. 5.16 ± 0.98 s post-FUS, respectively; Fig. 3I). Our findings indicate that FUS is capable of eliciting focal hemodynamic responses followed by modest contralateral responses. We hypothesize that the contralateral activity may be attributed to cortico-thalamo-cortical loop [22] or corticocortical communication [37]. Because of high spatiotemporal resolution of fUS imaging, we were able to temporally resolve the propagation of neurovascular activity from subcortical to cortical regions (Fig. 3E). The activation map with an early lag revealed CBV increases originated in the thalamus followed by the cortical activation that was overlapped with the activation by electrical stimulation evoking toe twitch. The focal-onset activation was co-localized with *in situ* brain displacement (Fig. 3B and Fig. 3E left), underlining that displacement imaging is capable of identifying the site of displacement-driven neuromodulation. Overall, the activated area included the primary somatosensory cortex and subcortex regions including thalamus, caudate putamen, and basal ganglia, which are known to be involved in motor function and sensory information processing (Fig. 3E, 6F, and 6G). We found a linear relationship with strong correlation between FUS-elicited CBV and EMG responses (r = 0.9041, p = 0.0002, *R*^2^ = 0.6876; Fig. 4F). Our findings indicate that fUS imaging successfully detect CBV that reflects neural activity causally linked with the motor responses induced by FUS.

Furthermore, we demonstrated highly lateralized limbic responses that correspond to lateralized thalamic activation (Fig. 5). The displacement map confirmed lateralized FUS targeting of the thalamus and showed co-localization with stronger neurovascular activation in the ipsilateral hemisphere (Fig. 5F), which is consistent with the aforementioned findings. Surprisingly, we observed lateralized movement of hindlimb (Fig. 5B and Supplementary Movie 5A and 5B), which was found to be ipsilateral to the FUS-evoked thalamic activation. Our findings on the lateralization on both neurovascular and motor responses may indicate that the ipsilateral limbic responses we observed were attributed to direct activation of subcortical motor circuit including thalamus and basal ganglia (Fig. 5F and 5G). The difference in the amplitude of hindpaw velocities between LTh and RTh FUS may be due to our motion tracking setup with a single camera that inherently cannot compensate for the recording angle. Nonetheless, FUS-evoked limbic responses showed a clear directional dependence on the hemisphere with the stronger subcortical neurovascular responses (Fig. 5C and 5D). Lateralization on both neurovascular and motor responses eliminates the possibility of neurovascular activation via indirect pathway associated with auditory or somatosensory confounds [12, 13] mediating FUS-evoked neurovascular and motor activation observed in this study. This is also supported by our findings that the EMG latency we found was significantly higher than an expected latency of a startle-like reflex, and no significant motor responses observed with off-target stimulation (Fig. 5E and Supplementary Movie 5C). In addition, the co-localization and correlation between brain displacement and neurovascular activation we consistently observed in this study precludes the contribution of the auditory or somatosensory confounds. Thus, we conclude that neurovascular and motor responses observed and reported in this study were attributed to the direct neurovascular/motor activation evoked by FUS via displacement-driven mechanism.

**Fig 1.**
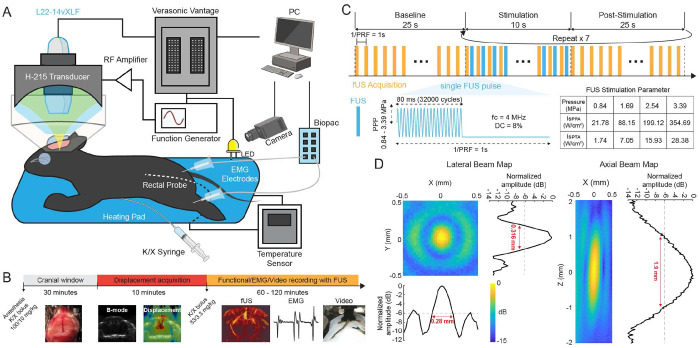
(A) Experimental setup for fUS imaging of displacement-guided FUS neuromodulation. (B) Experimental timeline. Displacement acquisition was followed by concurrent fUS/EMG/video recording with FUS stimulation. K/X: Ketamine/Xylazine. (C) fUS acquisition and FUS stimulation paradigm. The top panel depicts an interleaved sequence for fUS acquisition (orange) and FUS trigger (blue). The bottom panel depicts the FUS stimulation waveform. The table summurizes derated FUS pressure/intensity used in the study. (D) Hydrophone measurement of the lateral (left) and axial (right) beam profile of the H-215 FUS transducer at 4 MHz. The pressure amplitude along each axis was plotted in a logarithmic scale. The focal size in full-width half-maximum (FWHM) was denoted in red.

To induce neural excitation that successfully leads to muscle activation in hindlimb, hindpaw, and tail, we employed relatively high pressure that may not be considered as low-intensity focused ultrasound (LIFU). However, given the frequency of 4 MHz, the highest pressure of 3.39 MPa used in this study corresponds to 1.69 mechanical index (MI), which does not exceed MI safety limit of 1.9 based on ITRUSST guideline [38]. Thus, it is likely that the cavitation and/or mechanical risks are nonsignificant with the FUS parameters used in this study. To assess the safety of the FUS paradigm used in this study, we performed H&E staining (n = 1 animal; Supplementary Fig. S4) after sonicating the left hemisphere at 3.39 MPa with FUS stimulation paradigm as described in Fig. 1C. We did not observe any indication of brain damage or other irreversible effects, which is consistent with the safety results that we previously reported with longer pulse durations of 150-300 ms, but amplitude-modulated [4]. Importantly, we report a 1.72 °C increase in temperature induced by 10 FUS pulse train with FUS parameters used in this study (Supplementary Fig. S5, method established in [39]), which is significantly reduced compared to the previously reported temperature elevation of 4.6 °C [4] and 6.8 °C [14], more importantly, less than 2 °C safety limit [38]. Thus, it is likely that thermal risks are nonsignificant with FUS parameters used in this study as confirmed by the H&E staining analysis. We did not observe any comparable cumulative effect on FUS-evoked motor responses that was prevalent in the study [14]. Thus, we ascertain that FUS does not require significant heating or cumulative effects to elicit localized neurovascular activation and lateralized motor responses reported in this study. We believe a continuous pulse regime with relatively short pulse duration favors mechanical effect-driven neurovascular modulation and is much less susceptible to temperature-mediated vascular changes [40] that could confound neurovascular dynamics as neural correlates [41], thus better elucidating neurovascular responses that mediated by a mechanical effect of FUS.

We demonstrated the capability of transcranial fUS imaging to detect successful neurovascular modulation at the hippocampus, which was followed by behavioral changes that showed reduced anxiety (i.e. improved neuropsychiatric symptoms) based on the open-field test (Fig. 7A). The effect of FUS paradigms through neuromodulation or blood-brain barrier opening was evaluated on the capacity to ameliorate the anxiety, which is a characteristic symptom associated with Alzheimer’s disease. We found that FUS-N reduced anxiety significantly as compared to the sham group, with a close to significant reduction in the FUS-BBBO group (Fig. 7D). Additionally, the number of DCX+ cells, a stain used to identify cells undergoing neurogenesis, correlated with increasing time in the center zone (Fig. 7G, p = 0.0057), indicating that neurogenesis may contribute to the anxiety-relieving abilities of the FUS-BBBO. A similar trend is observed among the mice in FUS-N where the correlation is approaching significance (p = 0.0527). While the FUS-BBBO group presents a stronger correlation between DCX+ cells with the time in the center zone and an increased count, FUS-N significantly reduces anxiety compared to sham control. This difference may suggest another mechanism involved in the observed cognitive benefits. Although FUS-N failed to induce significant neurogenesis effect, notably, transcranial resting-state functional connectivity (group-averaged; n = 4 animals) measured 10 minutes after sonication showed significant increases in hippocampal connectivity with the retrosplenial, primary somatosensory, and visual cortices, compared to the sham groups (Supplementary Fig. S7). The hippocampal connectivity with other subcortical regions including striatum, globus pallidus, and thalamus also showed increasing trends, but did not reach statistical significance. We hypothesize that transcranial fUS-guided hippocampus FUS induced alteration of brain plasticity and hippocampal/sensory network modulation [42], leading to anxiety-related behavioral improvement that we observed in this study. While our findings demonstrated the ability of FUS to benefit anxiety, it requires further studies which may elucidate the relative importance of amyloid-beta clearance in diseased models through if FUS-N or FUS-BBBO as a therapeutic intervention may suffice to reduce Alzheimer’s disease related impairments [43]. A limitation of the behavioral study is the use of young, wild-type mice which are already undergoing neurogenesis. Future work will incorporate aged and diseased murine models to extrapolate the relationship between cognition and neurogenesis as a potential mechanism.

Our study was not devoid of limitations. The study was motivated to provide topographic mapping of a specific type of motor responses evoked by FUS, as our earlier study shed some light on [14]. However, we found it challenging to isolate only a single type of motor activation (e.g. isolated individual activation of the tail, hind-paw, or hindlimb). In this study, we rather observed a sum or combination of those muscle activations, which we demonstrated as bilateral toe twitch or lateralized limbic responses with tail movement. It may be largely because cortical and subcortical regions that topographically corresponds to the hindlimb and tail are not only anatomically adjacent but also functionally connected [44], and single-element FUS could be limited in the spatial resolution to effectively resolve and target a single topographic structure in small animal, especially in the axial direction as seen in our displacement map. Employing static [45] or dynamic holograms with a steering capability [18] will help confine FUS focus within the topographic structure of interest, yet necessitates consideration of the relationship between focal volume and neuromodulation efficacy [19]. Given the consistency between cortical activation evoked by FUS and electrical stimulation where both stimulation regimes evoked toe twitch in mice, it is plausible that FUS-evoked toe twitch was causally linked to neurovascular activity in the somatosensory cortex, whereas lateralized hindlimb responses and tail movement were associated with the subcortical activity.

Being analogous to isoflurane [46], ketamine induces vasodilation [47], leading to a lower contrast of CBV changes to the baseline, thus we anticipate that the FUS-evoked CBV responses we observed is indeed lower than the evoked responses in awake mice. To mitigate any confounding effect of anesthesia, combining awake setup for FUS-evoked motor responses [48] and recording fUS [49] would be intriguing. In this study, we used cranial window for higher fUS sensitivity to detect neurovascular activity that correlates with FUS-evoked motor responses, but our ongoing work includes transcranial displacement and fUS imaging to monitor transcranial FUS [50] to take account of the effect of skull on FUS neuromodulation and to translate our ultrasonic monitoring approach. In addition, we specifically aim to expand the capability of our ultrasoundbased mechanistic monitoring system into 3D using row-column addressed array [51] to investigate whole-brain CBV responses to FUS neuromodulation in mice (Supplementary Fig. S8). This will help elucidate the neuromodulatory effect of FUS on brain circuits or pathways beyond its focal, in-plane effects on neurovascular activity.

## 4 Conclusion

We report on neurovascular activity during motor responses evoked by DgFUS neuromodulation in K/X anesthetized mice. We found that DgFUS evokes localized, ipsilateral, and dose-dependent neurovascular responses followed by mild contralateral responses. The primary site of neurovascular activation was colocalized with *in situ* brain displacement map, exhibiting a peak CBV increase of 30.15 ± 15.47 % at 4.33 ± 0.51 seconds post-FUS at 3.39 MPa. Interestingly, we discovered toe twitch responses evoked by FUS, along with cortical CBV activation, comparable to those evoked by electrical stimulation. We demonstrate that a single 80-ms continuous FUS pulse successfully elicits target-specific motor responses in mice, including toe twitch and lateralized hindlimb responses with tail movement. We found a significant correlation between EMG and CBV responses (r = 0.9041, p = 0.0002) and EMG latency at 3.39 MPa was found to be 65.38 ± 8.52 ms post-FUS, significantly greater than the typical latency of the auditory reflex. By spatially translating DgFUS in the thalamus and switching hemispheres, we demonstrate lateralized hindlimb motor responses with directional dependence on lateralized neurovascular responses, highlighting highly target-specific responses with displacement guidance. In addition, we found that displacement correlates with both CBV and EMG responses (r = 0.9091, p < 0.0001 vs. CBV, and r = 0.7332, p = 0.0091 vs. EMG), which indicates a displacement-mediated mechanism by which FUS induces neurovascular and motor responses . Lastly, we demonstrate that transcranial fUS-guided FUS delivered to the bilateral hippocampus induces anxiety-related behavioral changes. The FUS-N group spent significantly longer time in the center zone compared to the sham group (p < 0.05), showing good agreement with increased functional connectivity in the FUS-N group. Our findings indicate that DgFUS can localize neurovascular activation and induce target-specific motor responses in a displacement-driven manner. It contributes to ongoing efforts to link displacement, neurovascular activity, and consequential motor responses evoked by FUS in mice. We anticipate that DgFUS will constitute a valuable tool that spans from basic neuroscience to therapeutic applications, allowing for more guided and informed neuromodulation using FUS with real-time feedback.

## 5 Methods

### 5.1 Animal preparation and surgery

All experiments and procedures in this study were performed in accordance with Columbia University Institutional Animal Care and Use Committee (Protocol # AC-AABF2550). Female C57BL/6J (Envigo; Indianapolis, IN, USA) ages 8 – 12 weeks were used in all experiments. In the study, a total of 22 wild-type mice (n = 6: fUS imaging with FUS for motor response experiment, n = 1: H&E histological evaluation, n = 1: temperature measurement, and for behavioral and connectivity experiments; n = 5: FUS-N, n = 5: FUS-BBBO, n = 4: sham) were used. Animals were anesthetized with 4% isoflurane for induction and 1-2% for maintenance in the behavioral and connectivity experiment. For the motor response experiment, bolus injections of a mixture of ketamine and xylazine were administered (Fig. 1B). The first bolus injection (ketamine 100 mg/kg and xylazine 10 mg/kg; 1:1 dilution in saline) was administered prior to craniotomy, and the second bolus injection (ketamine 33 mg/kg and xylazine 3 mg/kg; 1:1 dilution in saline) was administered prior to FUS stimulation. In all animals except mice used in behavioral and connectivity experiments, a large-window (9 mm × 5 mm) craniotomy was performed to achieve a higher signal-to-noise ratio (SNR) in displacement and fUS imaging. Body temperature was monitored and kept at 37°C with a heating pad with a rectal temperature sensor. Animals were subjected to toe pinching every 15 min to assess the depth of anesthesia. The additional bolus injection (ketamine 33 mg/kg and xylazine 3 mg/kg) was administered, if needed to maintain adequate anesthesia (i.e., no responses or very mild paw twitches when pinched).

### 5.2 Experimental setup

We employed a 128-element linear imaging transducer (L22-14vXLF; Vermon, France) for displacement and functional ultasound imaging. The single-element 4 MHz FUS transducer (H-215; Sonic Concepts, Bothell, WA, USA) was used to generate FUS. An ultrasound research system (Vantage 256 High-Frequency Option; Verasonics Inc., Kirkland, WA, USA) operated the imaging transducer and synchronized ultrasound imaging sequences and FUS trigger. The imaging transducer was inserted through a central opening in the FUS transducer, ensuring both transducers were coaxially aligned via a 3D-printed attachment. A 3D-printed collimator with an acoustic-permeable membrane was filled with degassed water to couple the transducers with the craniotomized brain.

### 5.3 Displacement-guided focused ultrasound (DgFUS) neuromodulation paradigm

We have established a 4 MHz FUS neuromodulation protocol for motor activation in K/X anesthetized mice. We used continuous FUS pulses with a pulse duration of 80 ms (320k cycles, *f_c_* = 4 MHz) and a sonication duration of 10 s at a pulse repetition frequency (PRF) of 1 Hz. After craniotomy, FUS was manually scanned over the window (with a scanning boundary of ML: −2.5 - +2.5, AP: −1.5 - 0, DV: 0.5 - 3 mm) to target the somatosensory cortex and thalamus until FUS elicits robust motor activation of hindlimb and/or toe twitch with tail movement. Once FUS was successfully located, where the mouse exhibited robust motor activation, displacement imaging was performed to confirm target engagement and quantify *in situ* brain displacement. CBV signals were also collected during FUS stimulation. To examine the dose-dependent effect of ultrasound, a derated peak positive pressure ranging from 0 (sham) to 3.39 MPa was explored. All pressures reported in this study were derated.

### 5.4 Displacement acquisition and processing

**Fig 2.**
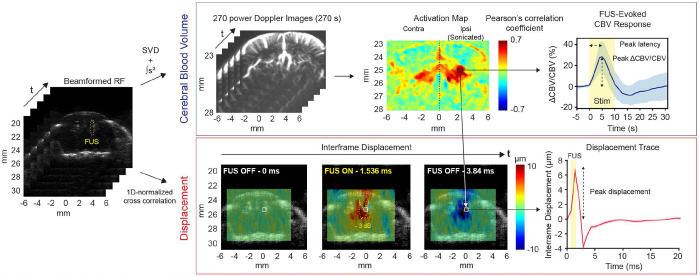
Processing pipelines for power Doppler and displacement and metrics used for quantification. (Top) Power Doppler images were obtained by accumulating SVD-filtered, beamformed RF data. In the example activation map, a black region of interest (ROI, shown in white in the displacement image) depicts a window with 5 x 5 pixels centered to the pixel with the highest correlation coefficient in the ipsilateral hemisphere. CBV and displacement signals were averaged within the same ROI to obtain mean CBV and displacement traces. In the example CBV trace, solid line and shadow represent mean and standard deviation, respectively (n = 7 stimulation periods). Yellow shaded area in the CBV plot depicts 10 s duration of a stimulation period. Peak latency and peak CBV changes were evaluated for quantification (dotted arrow). (Bottom) Interframe displacement was obtained by performing 1D-normalized cross correlation on the beamformed RF data. The yellow contour depicts −3 dB of displacement map at the displacement frame of 1.536 ms while FUS is active. Displacement trace calculated within the ROI (white) was used to determine peak displacement (dotted arrow). Yellow shaded area in the displacement plot depicts 1 ms FUS pulse duration.

**Fig 3.**
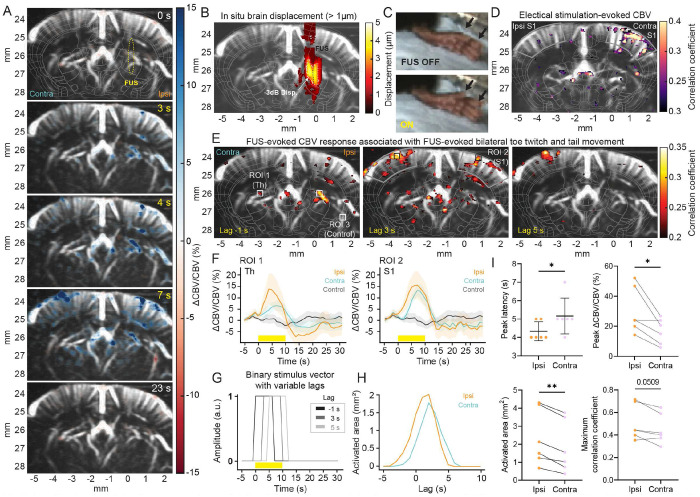
Localized and ipsilateral neurovascular modulation followed by contralateral neurovascular modulation associated with bilateral toe twitch and tail movement evoked by FUS. (A) CBV responses evoked by 2.54 MPa FUS at 0, 3, 4, 7, and 23 s after the onset of FUS. The yellow dotted area overlaid on the power Doppler image depicts the full-width half-maximum (FWHM) of FUS focus. (B) *In-situ* brain displacement (> 1 pm) map overlaid on power Doppler image. (C) Toe twitch response evoked by FUS. (D) Activation map with left hindpaw electrical stimulation that evokes the comparable toe twitch (1.5 mA, 7 ms pulse, 4 Hz, and 20 s duration). Gray contour depicts the primary somatosensory cortex (S1). (E) Activation maps with FUS stimulation based on Pearson’s correlation with −1 s (left), 3 s (center), and 5 s (right) lag. ROI 1 and 2 represent subcortical and cortical ROIs, respectively, whereas ROI 3 represents off-target ROI in the ipsilateral hemisphere. Gray contour was depicted as in (D). (F) Ipsilateral (orange) and contralateral (cyan) CBV responses at ROI 1 (left) and 2 (right) with control (gray; CBV response at ROI 3). Yellow square depicts 10 s FUS stimulation period. Solid line and shadow represent mean and standard error of mean, respectively (n = 7 stimulation periods from one animal). (G) Binary stimulus vector with variable lags that corresponds to (E). (H) The size of activated area as a function of lag. (I) Statistical results (mean ± std with data, n = 6 animals with FUS at 3.39 MPa) on peak latency and peak amplitude of ACBV/CBV, activated area, and maximum correlation coefficient from CBV responses at ROI 1. Two-tailed paired t-test was performed. Any statistical significance is presented (*p < 0.05, **p < 0.01).

**Fig. 4.**
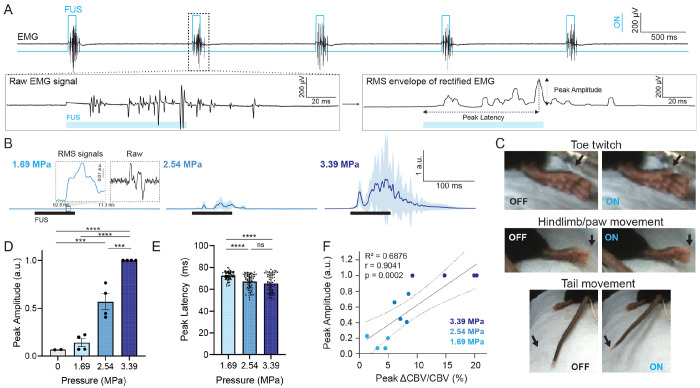
Electromyography (EMG) and locomotion behaviors evoked by 4 MHz FUS. (A) An example of EMG responses from tail (black) with 5 FUS pulses (blue) at 3.39 MPa. Peak amplitude and peak latency were obtained from root-mean-square (RMS) of rectified EMG signals (bottom right). (B) Normalized RMS of rectified EMG signals at 1.69 MPa (left), 2.54 MPa (center), and 3.39 MPa (right). The signals were normalized by the peak amplitude evoked by the highest pressure of FUS. In 1.69 MPa, representative EMG signals were depicted with FUS-evoked EMG peak RMS and raw signals magnified in the dotted box. In 2.54 MPa and 3.39 MPa, solid line and shadow represent mean and standard deviation, respectively (n = 69 stimulations). Black box underneath depicts FUS active duration. (C) Motor responses elicited by 3.39 MPa FUS observed throughout the study, including toe twitch (top), hindlimb/paw movement (middle), and tail movement (bottom). The black arrows in the left and right images in each row point to the same pixel to illustrate relative position of body parts. (D) Normalized peak EMG amplitudes with the different pressures (mean ± std with data, n = 4 animals). One-way ANOVA with Tukey correction for multiple comparions was performed (*** p < 0.001, **** p < 0.0001 ). (E) Peak Latency at 1.69, 2.54 and 3.39 MPa (mean ± std with data; n = 69 stimulations). One-way ANOVA with Tukey correction for multiple comparions was performed (”" p < 0.0001). (F) Correlation between peak CBV changes and EMG amplitude. A total of 12 CBV responses with different pressures were used in the analysis (n = 4 animals). Linear regression was performed (R^2^ = 0.6876, p = 0.0009), and a two-tailed nonparametric Spearman correlation was computed (r = 0.9041, p = 0.0002).

**Fig 5.**
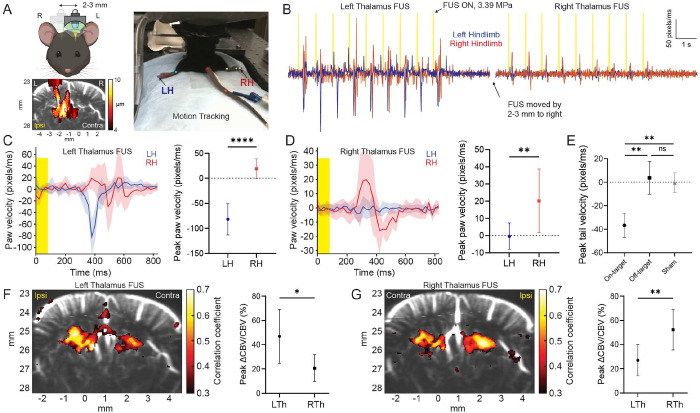
Spatially translated 4 MHz DgFUS drives co-localized thalamic activation and directional hindlimb motor activation. (A) Displacement-guided FUS physically translated to target left and right thalamus (left top). Example displacement map with −3dB contour (white dotted) in case of left thalamus FUS (left bottom). Motion tracking using DeepLabCut (right). Total of 9 points (3 points per body part) were labeled and tracked in the recorded video. The arrows point the tracked pixels used to calculate pixel velocities of the left and right paw. LH: left hindlimb, RH: right hindlimb. (B) Example time trace of paw velocities (pixels/ms) at left and right hind limbs with FUS stimulations targeting left thalamus followed by targeting right thalamus. To switch the hemisphere, FUS was displaced by 2-3 mm toward the other hemisphere. Blue and red lines depict paw velocities of left and right hind limbs, respectively, while yellow binary signals depict FUS ON and OFF periods. Averaged paw velocities in case of (C) left thalamus FUS and (D) right thalamus FUS. The yellow box depicts 80 ms FUS active duration. Solid line and shadow represent mean and standard deviation, respectively. Right panel depicts statistical results on peak paw velocities (mean ± std, n = 9 stimulations). A two-tailed unpaired t-test was performed. (E) Peak tail velocity in on-target, off-target (FUS moved forward by 0.5 mm), and sham (0 MPa) conditions (mean ± std, a total of 27 stimulations from from 3 animals). One-way ANOVA with Welch correction was performed for multiple comparisons (**p < 0.01 ). Representive neurovascular activation map (left) and statistical results on peak CBV changes (right; mean ± std, n = 7 stimulation periods) with (F) left thalamus FUS and (G) right thalamus FUS. A two-tailed unpaired t-test was performed. LTh: left thalamus, RTh: right thalamus. Any significance in the performed analyses is presented (* p < 0.05, ** p < 0.01, **** p < 0.0001 ).

**Fig. 6.**
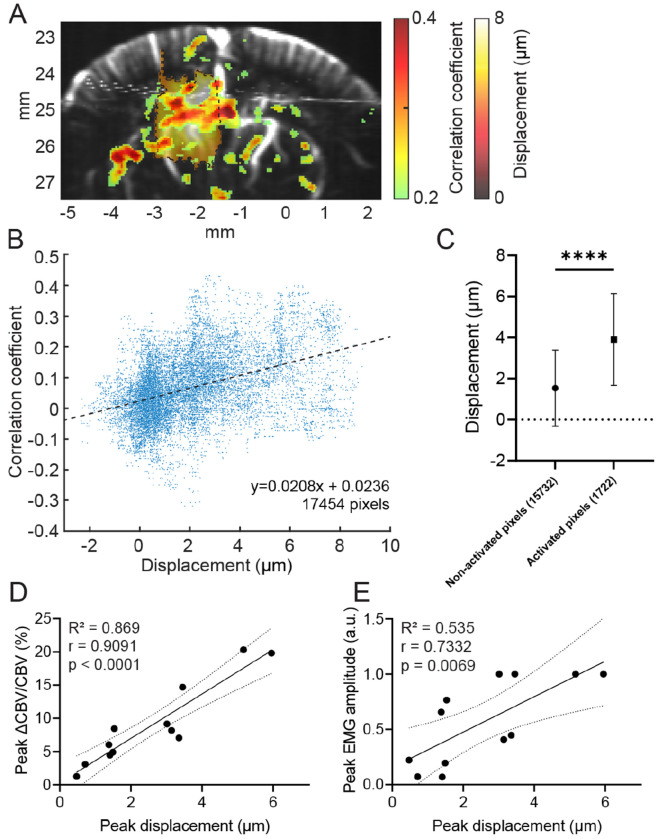
Displacement correlates neurovascular and motor responses. (A) Representative displacement-activation map with the left thalamus sonication. Brain displacement map with −3 dB contour (black dotted) was registered onto the activation map. (B) Scatter plot of correlation coefficient and displacement (17454 pixels). Correlation between displacement and correlation coefficient was evaluated by performing a two-tailed nonparametric Spearman correlation (r = 0.4166, p < 0.0001). Linear regression was performed (R^2^ = 0.174, p < 0.0001). (C) Comparison of displacement between two groups of pixels; one consists of 15,732 non-activated pixels, and the other consists of 1722 activated pixels. An unpaired t-test with Welch’s correction was performed to evaluate a statistical significance (**** p < 0.0001). Correlation between peak displacement and both (D) peak CBV change and (E) peak EMG amplitude. For the statistical analyses, a total of 12 CBV/EMG responses at 1.69, 2.54, 3.39 MPa were used in the analyses (n = 4 animals per pressure). Linear regression was performed (R^2^ = 0.869, p < 0.0001 vs. CBV, R^2^ = 0.535, p = 0.0069 vs. EMG), and a two-tailed nonparametric Spearman correlation was computed (r = 0.9091, p < 0.0001 vs. CBV, r = 0.7332, p =

**Fig 7.**
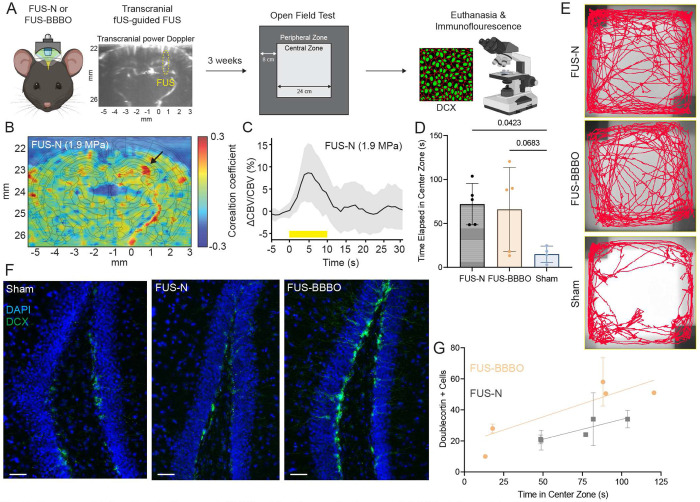
Transcranial functional ultrasound (fUS)-guided focused ultrasound (FUS) delivered to the bilateral hippocampus induces improvement in anxiety-related behavior. (A) Experimental timeline for behavioral and immunofluorescence analyses following FUS-N and FUS-BBBO. DCX: Doublecortin (B) fUS detects neurovascular modulation induced by FUS-N at 1.9 MPa on the hippocampus in the targeted (right) hemisphere. Correlation map revealed significant CBV activation at the right hippocampus (black arrow). (C) CBV response showed FUS-evoked CBV increases at the hippocampus with the peak amplitude of ~8.5%. The yellow square depicts FUS stimulation period. Solid line and shadow represent mean and standard deviation, respectively (n = 7 stimulation periods). (D) FUS-N treated mice exhibited significantly reduced anxiety in open field test (p = 0.0423). FUS-BBBO showed the same trend but did not reach statistical significance. One-way ANOVA with Tukey correction was performed for multiple comparisons (mean ± std with data, n = 5 animals). (E) Representative trajectory images of the open field test for FUS-N (top), FUS-BBBO (middle), and sham (bottom). (F) Immunofluorescence with DAPI and DCX. FUS paradigms increase DCX expression, with the greatest expression observed in the FUS-BBBO treated group. Scale bat denotes 50 μm. (G) Doublecortin positive (DCX+) cells correlate with the reduced anxiety (n = 5 animals). Linear regression was performed (FUS-BBBO: R^2^ = 0.6878 and p = 0.0057, FUS-BBBO: R^2^ = 0.3923 and p = 0.0527).

Prior to imaging and recording FUS-evoked responses, displacement imaging was performed to validate target engagement and to evaluate peak *in situ* brain displacement induced by FUS. We used 12 plane waves at angles evenly spaced between ± 3° and 1.5 cycles of the plane waves were transmitted at the carrier frequency of 15.625 MHz (imaging parameters shown in Supplementary Table 1). RF data for each displacement frame was collected every 0.768 ms, and a FUS pulse with a duration of 1 - 80 ms was triggered before the 3rd frame acquisition to generate displacement. After computing 1D normalized cross-correlation on compounded and beamformed RF, interframe displacement was obtained. The pixel-wise displacement was averaged within a window (5 x 5 pixels) centered on the pixel with the highest Pearson’s correlation (obtained from CBV analysis) to compute the averaged displacement (Fig. 2 bottom). The peak amplitude of the averaged displacement was used for statistical and correlation analyses.

### 5.5 Functional ultrasound (fUS) acquisition and processing

Ultrafast power Doppler acquisition and processing were performed as previously established in [4]. We used 13 plane waves at angles evenly spaced between ± 7° and 1.5 cycles of the plane waves were transmitted at the carrier frequency of 15.625 MHz (imaging parameters shown in Supplementary Table 1). Plane waves were sent at 19,500 Hz PRF and 500 Hz compounded PRF and 150 compound frames constitute a power Doppler image. To remove stationary tissue signals and reconstruct blood signals, the singular value decomposition (SVD) was used with a SVD cutoff of 30. Lastly, the reconstructed stack of beamformed RF was summed to obtain a final CBV image at 1 Hz functional framerate. We computed Pearson’s correlation between CBV signals and binary stimuli vectors to obtain an activation map. Pixels with the correlation greater than 0.2 (z > 3.3 and p < 0.001 with 270 samples) were considered as significant or activated by FUS. CBV responses from 7 stimulation periods were averaged out to obtain mean CBV responses. Peak CBV amplitude was used for statistical and correlation analyses.

### 5.6 Electromyography (EMG) acquisition and processing

Two pairs of unipolar electrodes (TE/S50718-001; Technomed Europe, Netherlands) were inserted into biceps femoris (hindlimb) and/or caudal (tail) muscle to collect compound muscle action potential (CMAP) evoked by FUS-elicited motor responses (recording parameters shown in Supplementary Table 1). We used data acquisition unit (MP150; Biopac, Goleta, CA, USA) with EMG interface module (EMG100C; Biopac, Goleta, CA, USA). Raw EMG signals were collected at 10 kHz sampling rate with a 0.5 to 5 kHz bandpass filter and a gain of 2000, then rectified and converted into root mean square (RMS) (Supplementary Fig. S1) [15]. To mitigate false peak detection from FUS stimulus artifacts, a detection window of 50 - 79 ms post-FUS was implemented. This range was informed by preliminary characterizations and used to isolate FUS-evoked EMG signals from FUS-onset and -offset electrical artifacts. Subsequently, findpeaks function in Matlab was employed to determine peak amplitude and latency. EMG responses from caudal muscle were used for statistical and correlation analyses.

### 5.7 Motion tracking of FUS-evoked motor responses

The paws of the right and left hindlimb tracked with DeepLabCut^™^ [25]. For each limbs, three points that spans from the edge of paw to ankle were tracked and the X and Y coordinates of the edge of paw were used in paw velocity analyses. The 2D-Euclidean distance was calculated from the movement in X and Y direction to obtain hindpaw velocity and the paw velocity was obtained per FUS pulse.

### 5.8 Statistical anaylses

All statistical analyses were conducted using Prism 9 (GraphPad; San Diego, CA, USA). To compare ipsilateral and contralateral neurovascular responses to FUS, two-tailed paired t-test were performed. To characterize normalized peak EMG amplitude and peak latency over pressures, one-way ANOVA with Tukey correction was performed for multiple comparison. To investigate correlation between motor and neurovascular responses, a two-tailed nonparametric Spearman correlation and linear regression were performed between EMG and CBV amplitude. To examine the lateralization in hindlimb movement, a two-tailed unpaired t-test was performed. One-way ANOVA with Welch correction was performed for multiple comparison between on-target, off-target, and sham. Finally, displacement between two pixel groups (activated and non-activated pixels) was statistically analyzed by an unpaired t-test with Welch correction. A two-tail nonparametric Spearman correlation was used to establish the relationship between displacement and both neurovascular and motor responses.

### 5.9 H&E staining anaylses

Mice were induced with a mixture of oxygen and 4-5% isoflurane and remained under heavy anesthesia throughout the procedure. They were transcardially perfused with cold, USP-grade 1xPBS for at least 5 minutes until the liver changed from red to brown in color. The brains were then extracted from the skull and placed in a solution of 4% paraformaldehyde for another 5 minutes. The brains were extracted and placed in a solution of 4 % PFA for 24 hours and then in 30% sucrose diluted in 1xPBS for at least 24 hr before sectioning. The brains were coronally sectioned at 40 um using ac cryostat (Leica CM1850, Leica Biosystems, Buffalo Grove, IL, USA).

### 5.10 Focused ultrasound-mediated blood-brain barrier opening

Focused ultrasound-mediated BBBO was performed with the regime previously established in our group [52]. A single-element, concave FUS transducer (center frequency: 1.5 MHz, focal length: 60 mm, diameter: 60 mm; Imasonic, France) and was operated at a Peak Negative Pressure of 450 kPa with a pulse repetition frequency of 10 Hz. A second single-element transducer V320, frequency: 7.5 MHz, focal length: 52 mm, diameter: 13 mm; Olympus NDT, Waltham, MA, USA) was aligned confocally with the FUS transducer and used for passive cavitation detection to monitor microbubble activity through the sonication. These transducers were mounted onto a 3D positioning system for targeting. Mice were anesthetized at 2-3% until induction and confirmed by no response upon a toe pinch and maintained anesthesia and maintained at 1-2%. The head was stabilized using a stereotactic apparatus and anesthesia.

### 5.11 Behavioral testing

The open field test was conducted using a 40 cm x 40 cm x 29.5 cm (L x W x H) opaque arena with an open top was centered under a Basler acA1300-60gm camera (Basler, Ahrensburg, Germany) inside of a noise canceling room (MDL 4848 S, Whisper Room Inc, Knoxville, TN 37919). An 8 cm-wide peripheral zone is defined along the edge of the arena (64% of the arena), and the remaining inner region (576 *cm*^2^) was defined as a brightly lit central zone (36% of the arena area). Each sub ject is placed mid-way along one of the arena walls, facing the wall at the start of the trial. The mouse is then allowed to explore the arena for 10 minutes, while Ethovision XT tracking software (Noldus Information Technology, Leesburg VA, 20176) recorded the total distance traveled, and the time elapsed in inner and peripheral zones in 5-minute intervals. The arena was cleaned with 70% Ethanol between subject trials. The time spent in the center is directly correlated reduced anxiety. Time spent in the center divided by the distance traveled permits normalizing by variations in locomotion.

### 5.12 Immunofluorescence analyses

The sections selected for staining corresponded to the area of opening. Free floating sections were washed 3x in PBS for 10 minutes and blocked for 1 hour in 1xPBS + 0.25% Triton (PBST) and 5% Normal Donkey Serum (NDS). They were then moved to the primary in PBST + 5% NDS and incubated at 4°C overnight using Doublecortin Antibody (C-18) (sc-8066, Santa Cruz Biotechnology). Sections were washed in PBST 3x and incubated in PBST + 5% NDS for two hours with Donkey anti-Goat IgG (H+L) Cross-Absorbed Secondary Antibody, Alexa Fluor TM 488 (A11055, Invitrogen). They were then washed 1x in PBS and then mounted using DAPI Fluoromount-G Mounting Medium. Imaging took place using the Olympus Bx61 microscope at 20x magnification. These images were then processed using ImageJ for qualitative assessment. Using a threshold, cells expressing DCX were manually counting using the Cell Counter plugin feature in image J. The cells on each hippocampus were counted and averaged per mouse. Statistical analyses were performed using an ordinary one-way ANOVA followed by Dunnett’s multiple comparisons test (alpha = 0.05) to compare each treatment group to the sham control in the behavioral test. Correlations between DCX cell counts and time spent in the center zone were assessed using simple linear regression for both groups. All statistical analyses were performed using GraphPad Prism (Prism 10 for Windows 64-bit, Version 10.5.0).

## Supplementary Material

This is a list of supplementary files associated with this preprint. Click to download.

SupplemantaryMovie3A.mp4

SupplementaryMovie5ERTh.mp4

SupplementaryMovie5DLTh.mp4

Supplementarydocument.docx

SupplementaryMovie5BRHL.mp4

SupplementaryMovie5ALHL.mp4

SupplementaryMovie4ALHL.mp4

SupplementaryMovie5COfftarget.mp4

SupplementaryMovie3BToetwitch.mp4

SupplementaryMovie4BRHL.mp4

**Supplementary information.** Supplementary movies and document are available online.

## Data Availability

The data for Figs. 3, and 5 used in this study are available at Github (https://github.com/sk5130/fUS-Disp-Motor-BehaviorPaper) Data for Figs. 4, 6, and 7 is available from the corresponding author upon request. Source data are provided with this paper.

## Code availability.

The code used for CBV analyses in this study is available at Github (https://github.com/sk5130/fUS-Disp-Motor-BehaviorPaper).
